# Ivermectin and malaria control

**DOI:** 10.1186/s12936-017-1825-9

**Published:** 2017-04-24

**Authors:** Satoshi Ōmura, Andy Crump

**Affiliations:** Graduate School of Infection Control Sciences, Kitasato University, 5-9-1 Shirokane, Minato-ku, Tokyo, 108-8641 Japan

**Keywords:** Ivermectin, Malaria, Feed-through insecticide

## Abstract

As the world begins to realize the very real prospect of eliminating malaria as a public health problem globally, the scientific community is acutely aware that novel and innovative new tools will be required if that lofty goal is to be accomplished. Moreover, the need for comprehensive, integrated products and interventions is being recognized in order for the critical ‘final steps’ toward elimination to be taken successfully. Failure to take these crucial last steps have dogged all past global disease elimination programmes, except for smallpox. The success of ivermectin in driving two of the most devastating and disfiguring neglected tropical diseases (NTD) to the brink of elimination has been well documented. The drug also bestows immeasurable non-target benefits, increasing the health and socioeconomic prospects of all communities where mass drug administration (MDA) has been carried out. Ivermectin kills a variety of parasites and insects, including the Anopheline vectors of malaria parasites. In view of long-standing MDA programmes, increasing attention is now being paid to the potential offered by re-formulating and re-purposing ivermectin to function as a feed-though mosquitocidal tool. This will provide a comprehensively beneficial weapon, for the anti-malarial armamentarium, as well as for probably improving the impact on existing target diseases. Prospects currently look highly promising, especially as the drug is already proven to be extremely safe for human use. However, for maximum impact, detailed analysis of various analogues of the unique ivermectin, as well as the parent avermectin compounds, will need to be undertaken. ‘Ivermectin’ comprises an imprecise mix of two compounds, both of which are potent anthelmintics. Yet recently, it has been confirmed that only the minor of the two component compounds is molluscicidal. Further structure activity relationship studies may well identify the analogue, analogues or combination thereof best suited for use in a concerted initiative to simultaneously tackle malaria and other NTD in poly-parasitized communities.

## Background

Expert comment on new research being published regarding the possibility of using ivermectin distributed to humans as a feed-through chemical to control blood-feeding mosquitoes.

## Main text

This Thematic Series contains a series of papers by Chaccour, Rabinowitz and Hammann [1–3] covering a possibly innovative anti-malarial mechanism, which will work synergistically with existing community-wide interventions to control or eliminate a handful of neglected tropical diseases (NTD). The discovery of ivermectin has already attracted the Gairdner Global Health award (2014) and the Nobel Prize in Physiology or Medicine (2015). Use of ivermectin as a feed-through anti-malarial has the possibility of helping to combat malaria, while simultaneously bestowing immeasurable public health and socioeconomic benefits upon millions of people in resource-poor communities throughout Africa and beyond.

Over the past 25 years or so, prodigious progress has been made in combating onchocerciasis through the distribution of donated ivermectin tablets to all members of communities where the disease occurs. Community-wide mass drug administration (MDA) of free ivermectin has driven onchocerciasis to the brink of elimination, with the innovative community-directed treatment (ComDT) system proving to be an ideal, cost-effective conduit for delivering integrated primary health services and tools—including the distribution of bed nets and home management of malaria—to remote, rural, underserved communities where the need for such interventions is greatest.

Ivermectin was the world’s first endectocide, capable of killing a wide variety of parasites and vectors, both inside and outside the body [4]. It is currently authorized to treat onchocerciasis, lymphatic filariasis, strongyloidiasis, scabies and head lice. In 2015, some 119 million of the 185.6 million Africans in need of ivermectin for onchocerciasis received it, with the prevalence of infection having fallen by over 70% since the intervention began [5]. The success of ComDT and ivermectin has resulted in the African Programme for Onchocerciasis Control (APOC) closing down, it being absorbed into a more extensive expanded special project for elimination of neglected tropical diseases (ESPEN). Launched in 2016, ESPEN is a 5-year project to expedite and accelerate the control and elimination of NTD amenable to preventive chemotherapy, namely onchocerciasis, lymphatic filariasis, schistosomiasis, trachoma and soil-transmitted helminths, using MDA of ivermectin and other donated drugs [6].

Much of the success in overcoming malaria has been due to control of *Anopheline* vector mosquitoes. Chaccour and others have shown that ivermectin that remains in the human bloodstream following a standard oral dose can kill blood-feeding *Anopheles* [7–11], as well as kill malarial parasites [12]. Consequently, as shown by the articles in this issue, ivermectin has significant potential of becoming a useful malaria transmission control tool, as proven by the establishment of the ‘Ivermectin Research for Malaria Elimination Network’ [13]. Moreover, current vector control depends substantially on the use of pyrethroids, yet mosquito resistance to pyrethroids has been increasing worldwide. The use of insecticide combinations in impregnated nets, possibly including ivermectin, offers the possibility of extending the usefulness of the nets, as well as slowing the development and spread of insecticide resistance, something which may merit investigation.

However, a note of caution needs to be sounded, which will necessitate further intensive investigation of ivermectin, its analogues and delivery systems. As worldwide attention on ivermectin and its potential steadily increases, it is becoming increasingly important to recognize that currently available ivermectin contains an imprecise mixture of two compounds [14]. The two component chemicals are extremely similar, compound 1 (ivermectin B1a) having an ethyl group at the C-26 position while compound 2 (ivermectin B1b) has a methyl group. ‘Ivermectin’ is routinely recognized as a mixture of 80% of 1 and 20% of 2. However, with the original manufacturer, Merck & Co. defining ivermectin as being composed of “at least 80%” of 1 and “not more than 20%” of 2 [15], there is room for considerable variance and, consequently, impact.

Increasingly, research is demonstrating that different ivermectin analogues can have differing bioactive characteristics. Compounds 1 and 2 are both potent anthelmintics, hence ivermectin’s effectiveness in initiatives to eliminate onchocerciasis and lymphatic filariasis. However, for example, a recent report has indicated that, when tested against the intermediate host snails in the schistosomiasis cycle, only one of the two constituents of ivermectin (the minor one) is molluscicidal [16]. It is thus likely that research into structure/activity relationships and evaluation of synthetic customized ivermectin analogues (or combinations thereof) is warranted and could prove extremely fruitful.

Furthermore, in humans, the high lipid solubility of ivermectin causes it to be widely distributed throughout the body, peak plasma concentration occurring some 4 h after oral dosing, a second peak at 6–12 h probably arising due to enterohepatic recycling, with the plasma half-life of ivermectin being around 12 h [17–19]. Consequently, the ability to kill blood-feeding mosquitoes dissipates relatively quickly after ivermectin dosing due to the swiftly declining plasma concentrations. It is, therefore, probable, as demonstrated in the papers in this journal, that slow-release formulations of ivermectin—not yet marketed or deployed—will be of enormous benefit, for killing internal worms and other parasites (internal and external) over extended periods, as well as in reducing the appearance of adverse side effects, and for repurposing ivermectin as an anti-malarial.

## Conclusion

The world is at a critical tipping point in the fight against malaria, if the progression from control to elimination is to be realized. Last year, the World Health Assembly endorsed the *Global Technical Strategy for Malaria 2016*–*2030,* which established ambitious goals, including a further 90% reduction in malaria incidence and mortality rates by the year 2030 [20]. In public health, malaria interventions generate premier returns on investment, simultaneously improving public health, helping to alleviate poverty, and improving social equity and sustainable development. In Africa, where the greatest disease burden exists, using ivermectin to help conquer malaria has the potential to boost further the immeasurable health and socioeconomic benefits that the drug has already delivered across the continent.

## Abbreviations

NTD: neglected tropical diseases
MDA: mass drug administration
ComDT: community-directed treatment
APOC: African Programme for Onchocerciasis Control
ESPEN: Expanded Special Project for Elimination of Neglected Tropical Diseases
